# Cognitive impairment among alcohol treatment service users in South Wales: an exploratory examination of typologies of behaviour, impairment, and service attendance

**DOI:** 10.3389/fpsyt.2024.1377039

**Published:** 2024-07-17

**Authors:** Nyle H. Davies, Julia Lewis, Bev John, Darren Quelch, Gareth Roderique-Davies

**Affiliations:** ^1^ Addictions Research Group, Faculty of Life Science and Education, University of South Wales, Pontypridd, United Kingdom; ^2^ Aneurin Bevan Specialist Drug and Alcohol Service, Newport, United Kingdom

**Keywords:** alcohol related brain damage, ARBD, alcohol dependence, addiction, cognitive impairment, cluster analysis

## Abstract

**Introduction:**

Alcohol dependence is a global issue with many negative consequences, including alcohol-related brain damage (ARBD). Assessment of the sociodemographic and cognitive characteristics of individuals with confirmed or suspected ARBD presenting to alcohol services warrants further investigation.

**Methods:**

This study retrospectively examined rates of cognitive impairment using Montreal Cognitive Assessment (MoCA) data from 300 adults who visited three alcohol support services. We demonstrate that 55.3% of the sample had significant levels of cognitive impairment. Females’ cognitive performance was disproportionately negatively affected by historical alcohol use relative to males.

**Results:**

The analysis identified four categories of participants, and the majority had a long history (+10 years) of alcohol use and were still actively drinking. Those taking part in active treatment for ARBD or practising abstinence demonstrated lower levels of cognitive impairment. Additionally, prior access to specialised ARBD care was associated with higher MoCA scores.

**Discussion:**

This research has identified a range of key service engagement, sociodemographic and cognitive characteristics that could be used to optimise support for those with alcohol dependence, whilst also highlighting some critical questions to be addressed in future research.

## Introduction

Harmful levels of alcohol consumption lead to significant personal, societal and economic burden (1). Individuals living with alcohol dependence often face increased morbidity, premature mortality (2) and a variety of healthcare provision disparities (3, 4). It is increasingly evident, but often still overlooked, that chronic heavy alcohol consumption is also linked to cognitive impairment. This is likely due to a lack of awareness in healthcare professionals and patients of the effects of alcohol on the brain. Alcohol Related Brain Damage (ARBD) is an umbrella term used to describe a group of conditions, all of which directly or indirectly result from the impact of chronic alcohol consumption on the brain. Symptoms of ARBD cognitive impairment (5), behavioural and emotional dysregulation (6–8), and difficulties with daily functioning (9–12).

Despite the evident need, there are numerous challenges to developing a clear treatment pathway to address ARBD (13). These include the complexity of the associated syndromes and underlying conditions; the lack of awareness and knowledge among healthcare practitioners (14); various personal, public and institutionalised barriers including stigma (15, 16); and the poor availability of effective education programmes (15). Whilst some clinical features of ARBD are well documented (17), there is a need for a greater understanding of patient engagement, cognitive and sociodemographic characteristics. For example, those with ARBD are not a homogenous group in terms of demographics, alcohol use history, service engagement and severity of cognitive impairment. Such differences in patient characteristics are likely to impact access to care and support. For example, patients with acute cognitive impairment may be viewed as intoxicated (18) or unmotivated and reluctant to engage with service staff (15, 19). Female patients are more vulnerable to the development of ARBD (20) and may experience a range of unique barriers to receiving treatment (21, 22). Furthermore, females are significantly less likely to access alcohol services compared with males (23). Older adults with alcohol use disorder are underdiagnosed and have been shown to receive inadequate support (24). However, there has been a 140% increase in those over the age of sixty presenting to hospital with amnesic symptoms associated with alcohol use (25). These examples highlight the need for a detailed understanding of how various characteristics such as gender, age and level of cognitive impairment may impact treatment access and how ARBD services and pathways should be developed.

Recent guidance describes the holistic assessment, including cognitive testing, of individuals engaging with alcohol services in order to identify those living with ARBD (13). The tools for neuropsychological assessment of individuals with alcohol related cognitive impairment have recently been reviewed (26). The Montreal Cognitive Assessment (MoCA) and the Addenbrooks Cognitive Examination III (ACE-III) are recommended as screening tools; both have demonstrated applicability in populations with ARBD (27, 28). The MoCA was initially developed in populations of mild cognitive impairment and mild Alzheimer’s disease (29) but has since been implemented for detection of cognitive impairment in a range of conditions (30). The MoCA can be utilised by healthcare professionals to assess memory (word recall), attention (serial subtraction and digit/letter lists), executive functioning and visuospatial ability (clock drawing, cube replication and path finding) language (word naming and phrase repetition), abstraction and orientation.

There is a growing body of evidence that highlights the utility of clustering approaches that examine person-level characteristics as a method of understanding the various typologies present in different populations (31–33). Cluster analysis has been applied to develop distinct typologies that inform clinical practice in the domain of alcohol withdrawal (34), gender difference in alcohol dependence (35), dual alcohol and marijuana use (36) and the relationship between emotion and interpersonal difficulties in alcohol dependence (7). This approach can provide a useful method of identifying key characteristics and subgroups within a specific population that can subsequently be used to inform and improve understanding of a given issue and has the potential to enhance the support provided.

Through implementing a clustering analytic approach, the present study aimed to explore the meaningful trends and associated characteristics of individuals with alcohol dependence presenting to alcohol services. The existence of any significant subgroups and relationships between these were explored with a view to develop knowledge that would facilitate engagement and development of alcohol treatment support.

## Methods

### Participants and procedure

The data were collected as part of a service evaluation at the Aneurin Bevan University Health Board examining cognitive burden in those with alcohol use disorder between June 2017 to August 2018. The service evaluation was approved by the Risk Review Panel at the Clinical Research and Innovation Centre at Aneurin Bevan University Health Board. The secondary analysis of this anonymised data was then approved by the Aneurin Bevan University Health board as well and the low-risk ethics committee at the University of South Wales. This resulted in final dataset which included all 300 patients that presented to the services between June 2017 and August 2018.

The dataset recorded alcohol use behaviour and a range of cognitive factors measured on the MoCA (version 7.1). The standard cut-offs described by the MoCA guidance were used to categorise the sample data. These were 18-25, which indicated mild cognitive impairment; 10-17, which indicated moderate cognitive impairment; and less than 10, which indicated severe cognitive impairment.

In addition to the MoCA scores, the following data items were also collected: demographics (age, gender), alcohol history (drinking history, abstinence history), drinking status (currently drinking, currently abstinent), previously identified as being cognitively impaired, and previous presentation with ARBD services.

### Services

The data were collected from three separate organisation which provided support for alcohol dependence, these included the Gwent Drug and Alcohol Service (GDAS), Gwent Specialist Substance Misuse Service (GSSMS) and the Cardiff and the Vale University Health Board Alcohol Care Team (ACT).

GDAS is a Third Sector Addiction Service commissioned to provide community treatment for people with alcohol use disorder. This includes psychosocial intervention (delivered individually or in groups) as well as medically managed alcohol withdrawal for patients at home who do not have complex physical or mental health needs requiring inpatient management. The MoCA was performed as part of the initial assessment on all new alcohol patients (most often as part of the first appointment). All assessment were carried out face-to-face at a community base local to the patient’s area. Referrals to GDAS are a mix of self-referrals and referrals from other agencies such as housing and primary care.

GSSMS is an NHS complex needs addiction service which provides medically managed inpatient alcohol withdrawal for patients presenting with complex needs (as identified using NICE (37) guidelines [CG115]) and for those with no suitable environment for home detoxification. As in GDAS, implementation of the MoCA was part of the established process for performing an initial assessment within the service. Referrals to GSSMS are from other agencies such as housing, primary and secondary care, and social services. This service does not accommodate self-referral.

ACT is an inpatient based alcohol liaison team. The service targets referred inpatients in a general hospital who had been admitted with an acute physical health problem which may or may not have been related to alcohol. The MoCA is performed during the first assessment of the patient.

### Data analysis strategy

IBM’s SPSS (version 29) was used to carry out all analyses. To simplify the data set (which includes variables such as alcohol consumption rate, abstinence rate, age, and gender), a two-step cluster analysis was applied to identify distinct groups of participants for comparison and contrast. This is achieved by ensuring that the intra-cluster distance (e.g., the distance between the data points within a cluster) is small and the inter-cluster distance (the difference between data points from different clusters) is large. The characteristics of these clusters are then explored to develop a clear understanding of the participant pool. Following this, one-way Bayesian ANOVA were used to examine the cluster level differences and other group level differences across the sample, additionally logistic regression was used to identify relevant predictive factors that could contribute to the understanding of the ARBD patient population.

### Missing data

Missing data were coded using the missing value function in SPSS prior to the Bayesian ANOVA and the logistic regression. However, due to the purposive sampling i.e. identification of inclusion through complete MoCA assessment the sample did not include any missing data.

Our two-step cluster analysis model requires a complete score on each of the included variables. Participants with incomplete data would therefore be excluded from cluster analysis in a case-wise fashion. However complete data on all the variables entered the two-step cluster analysis were available, as such there was no data loss regarding the development of the clusters.

## Results

MoCA results were included from 300 adults with a mean age of 45.5 (SD = 11.9), and 61.3% of the sample was male. The average duration of abstinence was low with a median of 0 (range 0 - 360 months). Among those who were practicing abstinence (N = 31) the mean length was 14.7 (Sd = 64.6), and the majority of the sample (N = 267) reported still consuming alcohol at the time of assessment (see **Table 1**).

**Table 1:** Participant demographics.

Age (Mean)	45.5 (SD = 11.9)
Gender	61.3% Male (N = 184)
Drinking abstinence in months (Mean/SD)	1.5 (20.9)
Drinking abstinence in months (Freq)	
0 months	87.7% (N = 269)
.5 months	1.7% (N = 5)
1 months	.0% (N = 15)
2 months	2.0% (N = 6)
3 months	.7% (N = 2)
15 months	.3% (N = 1)
46 months	.3% (N = 1)
360 months	.3% (N= 1)
Cognitive impairment	
Yes	22% (N = 66)
No	78% (N = 234
ARBD clinic presentation (Y/N)	
Yes	3.3% (N = 10)
No	96.7 (N = 290)
MoCA sub-domain score (total score available)	
Visuospatial (5)	3.8 (SD = 1.1)
Naming (3)	2.9 (SD = .35)
Attention (6)	5.1 (SD = 1.2)
Language (3)	2.0 (SD = .92)
Abstraction (2)	1.5 (SD = .67)
Recall (5)	2.8 (SD = 1.6)
Orientation (6)	2.8 (SD = 1.6)
MoCA total score (Mean/SD)	23.9 (4.3)

### Cluster analysis – cluster formation

A two-step cluster analysis model, allowing for the inclusion of both categorical and continuous data was selected. Eight variables were entered into the clustering model: age, MoCA total score, gender, previously identified as having cognitive impairment, attendance at a specific ARBD clinic, duration of drinking in years, abstinence in months, and whether the patient was drinking at the time of the assessment. Of the eight variables entered only six variables were identified as contributing to the cluster model (see **Table 2**). Due to homogeneity within MoCA total scores and age these variables did not contribute to differentiating patients between the clusters and so were non-contributary variables within the cluster model.

**Table 2:** Variables contribution to cluster model.

Variables	Contribution
Cognitive impairment (Y/N)	1.0
Currently drinking (Y/N)	0.94
Gender (M/F)	0.85
ARBD clinic attendance (Y/N)	0.25
Duration of drinking (Years)	0.05
Duration of abstinence (months)	0.03

Using Akaike Information Criterion as the clustering criterion a 4-cluster solution was identified. The cohesion and separation rating were good (0.6) indicating a robust cluster structure (-1 to 0.2 Poor/0.2 to 0.5 Fair/0.5 to 1 Good). The ratio of size was acceptable (2.96; accepted threshold of 3). The contribution of each variable to the cluster model can be seen in **Table 2** (ranging from 0 to 1) (see **Table 2**).

After examining the clusters’ demographic and participant scores, they were labelled based on the content of each cluster (e.g., predominantly male, rates of abstinence, presence of cognitive impairment).

The clusters were characterised in the following ways: Cluster A included long term impaired patients practicing abstinence; cluster B included untreated long term male patients; cluster C included untreated medium term female patients; cluster D included untreated, long term impaired patients (see **Table 3**). The characteristic labels selected provide a shorthand that describes each of the clusters; they are not necessarily a requisite characteristic for each of the participants included within the cluster. For example, the term ‘untreated’ was used to describe individuals who reported that they had not received treatment at an ARBD clinic; this does not indicate that the participant has not received any treatment for their issues relating to ARBD symptoms in another form or setting outside an ARBD clinic. As such cluster A included all the individuals who had attended an ARBD clinic, but the majority of those within cluster A had not attended an ARBD clinic.

**Table 3:** Cluster characteristics.

Cluster	Cluster description	Cog Impair^1^ (Y/N)	Currently drinking (Y/N)	Gender (M/F)	ARBD Treat^2^ (Y/N)	Dur. of drink^3^	Dur. of abstin.^4^
A	Long term impaired patients practicing abstinence	No	Yes	Both	No	Long	11
B	Untreated long term Male patients	No	Yes	M	No	Long	0
C	Untreated medium term female patients	No	Yes	F	No	Medium	0
D	Untreated long term impaired patients	Yes	Yes	Both	No	Long	0

(^1^cognitive impairment. ^2^attended ARBD specific clinic. ^3^Duration of drinking in years. ^4^Duration of abstinence in months).

### Cluster analysis – cluster characteristics

The number of individuals varied between the clusters: (see **Table 3**). Cluster A had 41 individuals, cluster B contained 121 individuals, cluster C had 85, and Cluster D had 53. (see **Table 3**).

Cluster A represented those who had 1) significant levels of impairment (mean MOCA score = 24), 2) a history of alcohol use spanning 13.4 years (SD = 10.4) and 3) were practicing abstinence (80% of individuals; mean duration = 11 months). This cluster was predominantly male (68.3%) and was the only cluster to include participants who had attended an ARBD clinic (24.4% of the cluster) (**Table 4**).

**Table 4:** Cluster characteristics.

	Clusters			
Characteristics	A	B	C	D
N	41	121	85	53
Age	49.1 (10.3)	44.5 (11.1)	45.9 (14.0)	44.1 (11.1)
Gender	68.3 Male	100% Male	100% Female	66% Male
Drinking abstinence (months)	11 (56.3)	0	0	.03 (.27)
Duration of drinking	13.4 (10.4)	14.2 (11.4)	9.1 (9.6)	15.2 (11.6)
Currently drinking	80.5% No	100% Yes	100% Yes	100% Yes
Cognitive impairment	68.3% No	100% No	100% No	100% Yes
ARBD clinic attendance	75.6% No	100% No	100% No	100% No
MoCA total (mean/SD)	25.1 (4.1)	24 (4.2)	23.7 (4.4)	23.2 (4.4)
MoCA risk category (N/%)				
No impairment	22/53.7	57/47.1	37/43.5	18/34
Mild Cog imp^1^	14/34.1	48/39.7	34/40	25/47.2
Moderate Cog imp	3/7.3	11/9.1	8/9.4	7/13.2
Severe Cog imp	2/4.9	5/4.1	6/7.1	3/5.7
MoCA sub-domains (mean/SD)				
Visuospatial	4.1 (1.2)	3.9 (1.1)	3.8 (1.2)	3.5 (1.1)
Naming	2.9 (1.2)	2.8 (.34)	2.8 (.47)	2.8 (.39)
Attention	5.3 (.89)	5.3 (1.1)	4.9 (1.4)	4.9 (1.4)
Language	2.0 (.87)	2.0 (.89)	2.0 (.90)	1.8 (1.0)
Abstraction	1.7 (.55)	1.5 (.65)	1.4 (.66)	1.4 (77)
Recall	2.8 (1.7)	2.8 (1.6)	3.0 (1.6)	2.4 (1.6)
Orientation	5.6 (.66)	5.4 (1.0)	5.5 (1.0)	5.4 (.77)

^1^cognitive impairment.

Cluster B represented the largest cluster; the characteristics were: 1) a history of consumption of 14.25 years (SD = 11.4), 2) lowest rates of abstinence (none of the patients were abstinent) and 3) no previous engagement with ARBD services. All the individuals in this group were male. The mean MoCA score for the cluster was 24.2 (SD = 4.2); none of the sample had been identified previously as having a cognitive impairment.

Cluster C represented 1) female participants, 2) who were not practicing abstinence and, 3) had been consuming alcohol for an average of 9.1 (SD = 9.6) years. The participants in this cluster were not identified previously as having a cognitive impairment and none of the sample had accessed ARBD specific services. Interestingly, while this cluster had the shortest history of alcohol consumption compared to the other clusters, their rates of impairment were comparable with the other clusters, with a mean MoCA total score of 23.7 (SD = 4.4).

Finally, Cluster D represented those with 1) the longest history of alcohol consumption (mean = 15.2 (SD = 11.6) years), 2) high rates of cognitive impairments (mean MoCA score of 23.2 (SD = 4.4)) and 3) low rates of abstinence. While the participants of this cluster had not engaged with an ARBD specific service, 100% had been previously identified as having a cognitive impairment.

### Cluster membership and severity of cognitive impairment

The relationship between the clusters and MoCA scores were explored, this indicated that there was no significant difference between the clusters regarding MoCA total scores (F (3, 2956) = 1.757, p.195. Bayesian factor = .003). Additionally, the clusters did not differ significantly when examining the individual domains of the MoCA.

### Age and ARBD service attendance

A significant age difference between those who had, and had not, attended an ARBD clinic was observed (F1, 299) = 9.092, p = .003. Bayesian factor = 3.9). The mean age for those who had attended was 56 (SD = 14.0; (95% credible interval (CI) 49.2 to 63.9) while the mean age for those who did not was 45.1 (SD = 0.4; 95%CI 43.7 to 46.4).

### Predictors of service attendance

A logistic regression was used to examine the predictive power of various variables concerning ARBD service attendance. Variables in the predictive model were age, gender, abstinence in months, drinking in years, MoCA total scores and whether the participant was drinking at the time of the assessment. Age and MoCA total scores were significant predictors of ARBD clinic attendance (X^2^ = 27.08, df = 6, P <.001); the Hosmer and Lememshow test was non-significant (X^2^ = 7.072, df = 8, P <.529) further supporting the model’s predictive ability. Gender, whether the participant was currently drinking, length of abstinence and duration of drinking (in years) were non-significant. The model accounted for 34% of the variability in ARBD clinic attendance and correctly classified 97.1% of the cases. Increased age and MoCA scores were associated with a greater likelihood of having attended an ARBD clinic.

### ARBD service attendance and severity of impairment

A significant difference between those who had, and had not, accessed an ARBD service (F(1, 298) = 8.213, p =.004, Bayes factor = 2.6) was observed. The mean MoCA score for those who had attended an ARBD clinic was higher [27.8 (1.8)] compared with those who had not (23.8(CI95% 25.1- 30.4, and 23.3-24.3), respectively).

### Service type and cognitive impairment

A statistically significant difference between cognitive impairment and the service types (ACT, GSSMS, ABSDAS) (F (2,297) = 16.112, P = <.01. Bayes Factor = 13835) was observed. Participants in the GSSMS service had significantly higher MOCA scores (27.2, SD = 3.4), when compared with ACT (23.5, SD = 3) and ABSDAS (23.3, SD = 4.4; mean difference compared to ABSDAS (3.8, p <.01) and ACT (3.6, p<.01) (see **Table 5**).

**Table 5:** Frequency of cognitive impairment across service type.

Service type	No cognitive impairment	Mild cognitive impairment	Moderate cognitive impairment	Severe cognitive impairment
ACT	14 (34.1/10.4)^1^	22 (53.7/18.2)	5 (12.2/17.2)	0
GSSMS	36 (80/26.9)	7 (15.6/5.8)	1 (2.2/3.4)	1 (2.2/6.3)
ABSDAS	84 (39.3/62.7)	92 (43/76)	23 (10.7/79.3)	15 (7/93.8)
Total	134 (44.6%	121 (40.3%)	29 (9.6)	16 (5.3%)

ACT, Cardiff and the Vale University Health Board Alcohol Care Team; GSSMS, Gwent Specialist Substance Misuse Service; ABSDAS, Aneurin Bevan Specialist Drug and Alcohol Service. ^1^frequency (percentage within service type/percentage within MoCA category).

### Service type and abstinence

A significant difference in length of abstinence between the services (F (2, 297) = 3.736, p = .02) was observed. The Bayes factor of.131 suggest that the difference between the groups is likely not to be clinically meaningful.

## Discussion

The present study has identified clinically relevant trends within a dataset of those presenting to alcohol support services. Due to the breadth of the data, a data reduction technique (cluster analysis) was employed to better understand the various characteristics and trends within the sample.

The cluster analysis identified four distinct typologies of participants who shared multiple traits relating to alcohol history and consumption, MoCA scores, abstinence, and treatment status. While the cross-sectional nature of the data does not allow for statements regarding the causal relationships between the included variables, the findings do highlight the potential effect specialist ARBD service could have on addressing cognitive impairments in those with alcohol dependence.

Finally, the utility of the MoCA was also highlighted as an efficient method of screening and patient monitoring which, when combined with specialised service expertise, could represent a meaningful pathway for supporting patients with ARBD. This study contributes to our understanding of the various sub-population of those with alcohol dependence and has implications for future research and care.

The analysis has identified the possible benefit of specialised services aimed at addressing ARBD. Patients who engaged with specialist ARBD service had lower rates of cognitive impairment and were grouped within a cluster among which the greatest length of abstinence was observed. As previously stated, ARBD is a non-progressive condition with a reversible component (13), and early identification can lead to a reduction and improvement in cognitive symptoms (38, 39). This may suggest that the unique skills and expertise within the GSSMS may support this reversibility, as demonstrated by abstinence and MoCA scores. However, given the cross-sectional nature of the data, it is difficult to isolate a causal relationship between these factors. Nevertheless, ARBD is common among AUD but often overlooked, as such there is a need for greater awareness and effective evidence based clinical pathways to address this largely hidden problem (40).

Another important aspect identified by this research is the value of the MoCA as a means to identify ARBD and as a method of patient monitoring which facilitates research and assessment. The MOCA has been identified as having high levels of sensitivity and specificity (27) and has been shown to be effective within AUD populations (41). Additionally, the MoCA is especially effective at identifying mild cognitive impairment (42), which, combined with its ease of administration, highlights it as an effective tool for early identification of ARBD (26).

A significant proportion of the sample reported high rates of alcohol consumption over many years. A small percentage of the sample was abstinent and very few had been abstinent for any significant period (longer than two years). In addition to high consumption rates, 55.3% of the sample showed some level of cognitive impairment as measured on the MoCA. Whilst not significant, these deficits were less evident in those receiving treatment or those practising abstinence. Recent studies report those who received alcohol treatment tended to show significantly lower rates of cognitive impairment. For example, Seddon et al. (39) demonstrate that alcohol treatment was associated with significant reductions in rates of cognitive impairment among older adults with long drinking histories. This further emphasises the importance of treatment and support to reduce harm in high-risk samples.

Gender differences were uncommon within the sample, but men and women differed in number of years of drinking and the onset of cognitive impairment. Whilst no gender differences in total MoCA scores between men and women were observed our data does suggest that women experience comparable levels of cognitive impairment following fewer years of harmful drinking behaviours when compared with their male counterparts. This may represent greater risk of developing alcohol-related cognitive impairments among women. Various lines of evidence suggest that women are significantly more likely to suffer alcohol-related health problems such as kidney and liver damage (43, 44); these trends may also apply to cognitive impairment (45). This may relate to sex differences in the absorption, metabolism (46) and biodistribution (47, 48) of ethanol. This is especially important when examining the growing parity between men and women regarding rates of alcohol use disorders (49).

Our data suggest that the level of cognitive impairment and an individual’s age were significant predictors of whether they would attend a ARBD clinic. Young (18-29) and older adults (60+) are underrepresented in the sample, suggesting they are less likely to attend alcohol services, with a similar pattern being observed in the wider UK population (50). Whilst this underrepresentation of younger adults might be expected because they would have had less time for alcohol dependence and ARBD to progress, evidence suggests that early identification of harmful levels of alcohol consumption is inadequate in this age group resulting in fewer young people in treatment. Similarly, older adults are more likely to go undiagnosed or misdiagnosed across various medical issues (51), including alcohol use (52). A report produced for AlcoholChange (53) demonstrate that practitioners could not identify signs and symptoms of harmful alcohol use in older adults. Additionally, some practitioners stated that older adults could not change problem drinking behaviour due to their age and that it could be construed as unfair to remove their “last pleasure in life” (53). These identification issues are further compounded by evidence suggesting that older adults rarely seek treatment for alcohol issues (54) and that alcohol use can lead to premature ageing of the brain (55). The present sample is limited to participants from three specific services. As such, it is evident that the impact of age on identification and treatment requires further examination.

We report data reflecting outcomes from the first cognitive assessment performed in individuals engaged in each service. Repeat measures were not available. Whilst the decision to perform cognitive assessment would, in part, be guided by medical history and screening guidance (56) upon engagement in a service, clinical need and availability of expertise may also prompt assessment. As such the timepoint at which cognitive assessment occurred during a patient’s recovery journey may vary. For example, some may have been engaged with a service for a number of months or sessions before the need for cognitive assessment was identified.

This exploratory study has identified several critical questions that should be addressed in future research. Only a relatively small portion of those with alcohol dependence had accessed specialised care that addressed ARBD. As such, examining and addressing the personal and organisational barriers to development and access to these services is vital. Additionally, the extent to which these barriers are mediated by demographic factors such as age, gender and ethnicity, and behaviour factors such as consumption warrants further investigation. Gender was identified as a meaningful factor when examining those with alcohol dependence. The present study noted that female participants reported a pattern of accelerated harm compared to males. On average, female participants had a significantly shorter history of alcohol consumption but showed comparable rates of cognitive impairment. As such, efforts should be made to pro-actively identify this at-risk group with a view to facilitating earlier diagnosis and intervention. Finally, this research has shown that those who have attended ARBD clinics and were practising abstinence tended to show less cognitive impairment when compared to those who did neither. While this evidence supports the critical nature of such factors in addressing ARBD, there is room for further examination. For example, what is the timeframe or rate of recovery, and how are these mediated by various patient, intervention and service delivery factors?

In summary, this research has three key findings: 1) that those attending alcohol services clustered into discrete typologies based on certain demographic and alcohol use history characteristics, 2) that those attending specialist ARBD services were more likely to engage in abstinence, which combined with specialist input could lead to improvements in cognitive performance, and 3) that female patients with relatively shorter alcohol use histories compared to males, were equally as cognitively impaired, suggesting the increased vulnerability of this group to the effects of alcohol on the brain.

## Data availability statement

The data analysed in this study is subject to the following licenses/restrictions: Secondary data analysis of data owned by ABUHB. Requests to access these datasets should be directed to julia.lewis3@wales.nhs.uk.

## Ethics statement

The studies involving humans were approved by University of South Wales Psychology Ethics Sub-Panel. The studies were conducted in accordance with the local legislation and institutional requirements. Written informed consent for participation was not required from the participants or the participants’ legal guardians/next of kin in accordance with the national legislation and institutional requirements.

## Author contributions

ND: Data curation, Methodology, Writing – original draft, Writing – review & editing. JL: Data curation, Writing – original draft, Writing – review & editing. BJ: Conceptualization, Data curation, Supervision, Writing – original draft, Writing – review & editing. DQ: Writing – original draft, Writing – review & editing. GR-D: Conceptualization, Project administration, Supervision, Writing – original draft, Writing – review & editing.
